# Detection of Plasmid-Mediated Resistance to Metronidazole in Clostridioides difficile from River Water

**DOI:** 10.1128/spectrum.00806-22

**Published:** 2022-08-11

**Authors:** Alois Cizek, Martina Masarikova, Jan Mares, Marie Brajerova, Marcela Krutova

**Affiliations:** a Department of Infectious Diseases and Microbiology, Faculty of Veterinary Medicine, University of Veterinary Sciences Brno, Brno, Czech Republic; b Department of Zoology, Fisheries, Hydrobiology and Apiculture, Faculty of AgriSciences, Mendel University, Brno, Czech Republic; c Department of Medical Microbiology, Charles University, 2nd Faculty of Medicine and Motol, University Hospital, Prague, Czech Republic; Institut National de Santé Publique du Québec

**Keywords:** wastewater treatment plant, surface water, plasmid-bound metronidazole resistance, *erm(B)*, ribotyping, MLST, antimicrobial resistance

## Abstract

Clostridioides difficile is one of the most important human pathogens. The identification of its possible sources is important for the understanding of C. difficile infection (CDI) epidemiology. A total of 16 water samples from wastewater and surface water in South Moravia in the Czech Republic and 82 samples of fish and gulls were collected between May and July 2019. C. difficile isolates were cultured by direct plating and after enrichment on chromogenic media. Susceptibility testing to eight antimicrobials was performed by Etest. C. difficile isolates were characterized by ribotyping, multilocus sequence typing, multilocus tandem repeats analysis, and toxin gene detection. Samples from fish and gulls were C. difficile negative; a total of 15 C. difficile isolates from 8 out of 16 water samples were cultured (6 out of 14 surface water samples yielded 6 isolates, and 2 out of 2 wastewater samples yielded 9 isolates). Direct plating was culture positive in 6 out of 16 samples (12 isolates), and enrichment culture was positive in an additional 2 out of 16 samples (3 isolates). Twelve different ribotyping profiles and 14 sequence types of clades 1, 4, and 5 were identified. Five isolates did not carry genes for toxins, and eight isolates carried genes for toxins A and B; the remaining two isolates (RT078) carried the genes for toxins A, B, and binary. All C. difficile isolates were susceptible to amoxicillin, moxifloxacin, tetracycline, and vancomycin and resistant to ciprofloxacin. A high level of erythromycin resistance (>256 mg/L) was detected in eight isolates. Clindamycin resistance was found in 14 isolates, 6 of which showed a high level of resistance (>256 mg/L) and carried *ermB*. Surprisingly, one isolate (RT010, ST15) showed resistance to metronidazole (12 mg/L) with the presence of the plasmid pCD-METRO. In conclusion, a diverse spectrum of C. difficile strains was found in wastewater and surface water. A recently discovered plasmid-bound resistance to metronidazole was detected in C. difficile from the surface water sample.

**IMPORTANCE** The combination of direct plating and culture after enrichment was used in order to gain a spectrum of C. difficile ribotypes present in the water samples. Toxigenic C. difficile ribotypes detected in surface water and in wastewater treatment plants overlapped with those derived from patients with CDI and/or animals. Importantly, a recently discovered plasmid-mediated resistance to metronidazole, a drug used for the treatment of CDI, was detected in C. difficile from river water.

## INTRODUCTION

Clostridioides (Clostridium) difficile is a spore-forming, Gram-positive anaerobic bacterium and is the leading pathogen of health care-associated gastrointestinal infections (1–3). Recently, an increase in C. difficile infections (CDIs) and high rates of asymptomatic carriage of C. difficile in the community have been recognized, suggesting that C. difficile reservoirs exist outside hospitals (4, 5).

One Health concept studies that focus on C. difficile clearly show that the intestine of healthy animals and humans may be an important reservoir of C. difficile (4, 6, 7) and that resistant spores facilitate its subsequent spread and survival in different types of environment. C. difficile spores are thus considered to be a major vehicle of transmission in humans, and a contaminated environment may play a role in an increased C. difficile occurrence in the community (5).

C. difficile has been recovered from food, soil, compost, river water, and municipal wastewater (8–10). Effluent and biosolids from wastewater treatment plants (WWTPs) are especially regarded as a potential source for the transmission of C. difficile from urban areas into the surface water through the feces of humans and animals (11). Therefore, contaminated surface water by effluent, downstream of the WWTPs, may allow the dissemination of C. difficile spores that can subsequently become the source for colonization of humans and animals. The presence of C. difficile spores in surface water can be also associated with C. difficile contamination in retail fish and seafood (12).

It is unknown if environmental contamination by C. difficile spores can be the direct source of community-acquired CDI. However, the significant overlap between human and environmental C. difficile ribotypes, despite their genetic diversity overall, has been documented (8).

**FIG 1:**
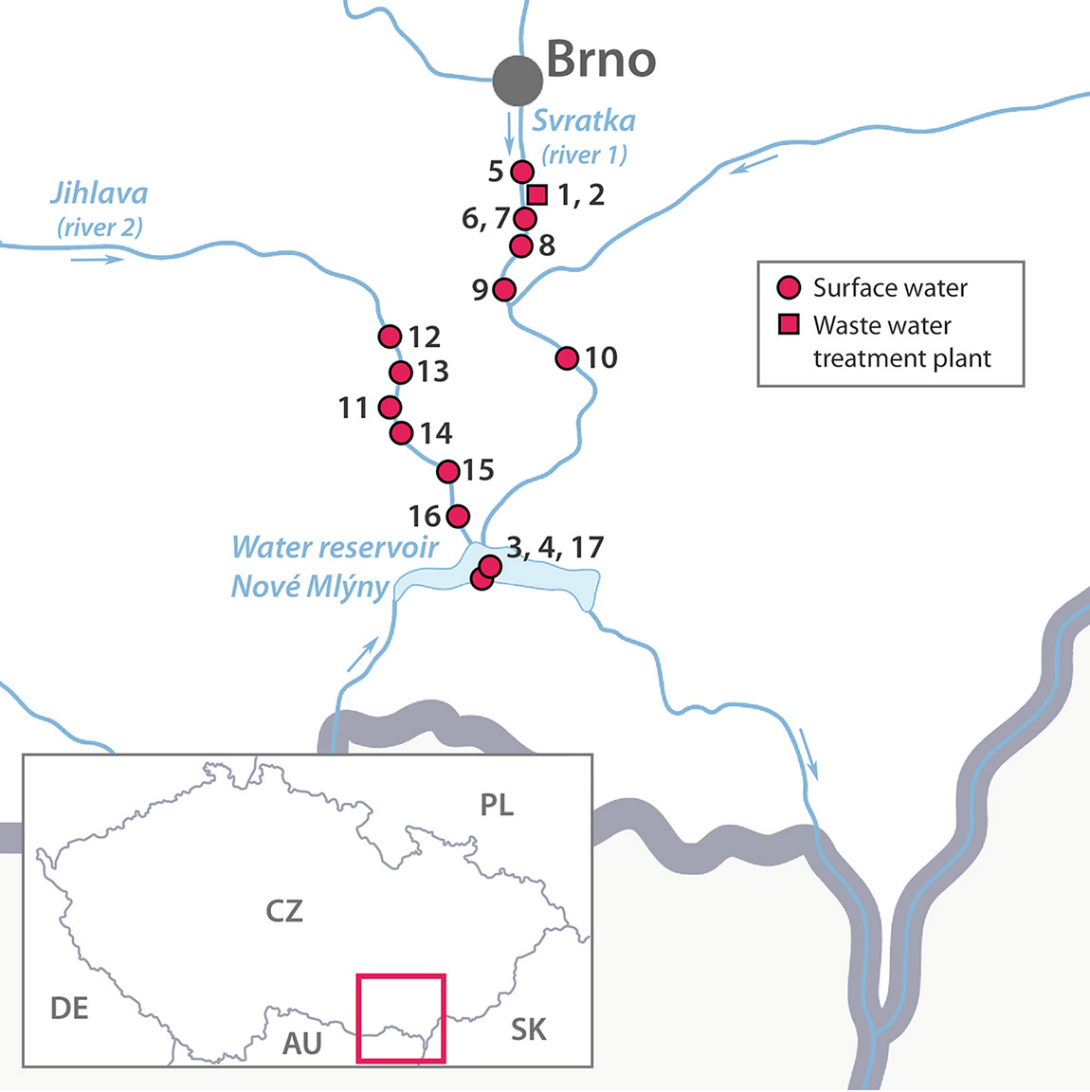
Sampling sites used in this study.

This study aimed to investigate the occurrence of C. difficile in surface water from the South Moravia river basin in connection with its occurrence in the effluent from the Brno wastewater treatment plant (Fig. 1). Subsequently, the presence of C. difficile was investigated in samples from fish and gulls living in the downstream reservoir.

## RESULTS

A total of 16 water samples (wastewater treatment plant, *n* = 2; lake, *n* = 2; river 1, *n* = 6; river 2, *n* = 6) and 82 samples from fish (*n* = 37) and gulls (*n* = 45) were collected between May and July 2019 (Fig. 1). Overall, 15 C. difficile isolates from 8 out of 16 water samples were cultured, including WWTP (9 isolates from 2 samples), lake (1 isolate from 2 samples), and rivers (5 isolates from 12 samples) (Tables 1 and 2). Direct plating was culture positive in 6 out of 16 samples (12 isolates), and enrichment culture was positive in an additional 2 out of 16 samples (3 isolates) (Table 1). Culture positivity of surface water was 6 out of 14 (42.9%) and of WWTP was 2 out of 2 (100%). The samples from fish and gulls were C. difficile culture negative.

**TABLE 1:** Overview of samples and culture results in the study^*a*^

Type of sample	Sampling date	Sampling place (GPS coordinates)	Map no.	Type (no.) of isolates detected by:		No.(s) of C. difficile isolates, no. of DNA
				Direct plating procedure	Enrichment and ethanol procedure	
Wastewater	13 May 2019	WWTP outflow (49°07′28.1″N, 16°37′44.5″E)	1	C. difficile (7)	ND	5852–5857, 5866
Wastewater	21 May 2019	WWTP outflow (49°07′28.1″N, 16°37′44.5″E)	2	ND	C. difficile (2)	5858, 5859
Surface water	25 May 2019	Nové Mlýny Reservoir (48°53′34.7″N, 16°36′20.7″E)	3	Clostridium butyricum	ND	
Surface water	3 June 2019	Nové Mlýny Reservoir (48°53′34.3″N, 16°36′19.0″E)	4	C. difficile	Clostridium clostridioforme	5860
Surface water	3 June 2019	Svratka River (pre-WWTP) (49°07′56.0″N, 16°37′38.5′E)	5	C. difficile	Clostridium celerecresceus	5861
Surface water	4 June 2019	WWTP, downstream Svratka River (49°07′51.9″N, 16°37′47.5″E)	6	Alistipes finegoldii	Clostridium hathewayi	
Surface water	4 June 2019	WWTP, downstream Svratka river (49°07′27.4″N, 16°37′36.1″E)	7	ND	C. butyricum	
Surface water	5th June 2019	Svratka River (49°05′34.2″N, 16°37′11.7″E)	8	ND	C. butyricum	
Surface water	5 June 2019	Svratka River (49°02′54.3″N, 16°36′44.3″E)	9	C. difficile	C. butyricum	5862
Surface water	5 June 2019	Svratka River (49°00′40.1″N, 16°39′16.0″E)	10	C. difficile	C. clostridioforme	5863
Surface water	6 June 2019	Jihlava River (48°59′30.9″N, 16°31′09.3″E)	11	C. perfringens	C. difficile	5864
Surface water	6 June 2019	Jihlava River (49°01′31.0″N, 16°31′13.0″E)	12	C. difficile	C. butyricum	5865
Surface water	6 June 2019	Jihlava River (49°00′32.8″N, 16°31′53.6″E)	13	ND	C. butyricum	
Surface water	10 June 2019	Jihlava River (48°58′54.5″N, 16°32′03.7″E)	14	C. butyricum	C. butyricum	
Surface water	10 June 2019	Jihlava River (48°57′25.8″N, 16°34′20.2″E)	15	C. butyricum	C. butyricum	
Surface water	10 June 2019	Jihlava River (48°55′40.4″N, 16°34′21.0″E)	16	C. butyricum	ND	
European herring gulls (*n* = 45)	25 May 2019	Gulls colony (48°53′34.7″N, 16°36′20.7″E)	17		ND	
Caught fish (*n* = 37)	10 July 2019	Nové Mlýny Reservoir (48°53′05.0″N, 16°36′00.7″E)	17		ND	

a WWTP, wastewater treatment plant; ND, not detected.

**TABLE 2:** Characterization and antimicrobial susceptibility of Clostridioides difficile isolates in this study

Sample type (sample no.)	No. of DNA samples	MIC (mg/L) of:								*tcdA*	*tcdB*	*cdtA/B*	RBT	ST	Clade
		CIP (>4)	CLI (≥8)	ERY (≥8)	AMX (≥16)	TET (≥16)	VA (>2)	MXF (>4)	MTZ (>2)						
WWTP (1c)	5852	>32	24	3	0.5	8	0.75	1.5	0.38	Neg	Neg	Neg	629	109	4
WWTP (1b)	5866	>32	>256^*a*^	>256^*a*^	0.5	0.023	0.5	2	0.25	Neg	Neg	Neg	010	15	1
WWTP (1d)	5853	>32	12	6	0.125	0.016	0.75	0.75	0.25	Pos	Pos	Neg	011	325	1
WWTP (1f)	5854	>32	>256^*a*^	>256^*a*^	0.19	0.75	0.125	1.5	0.25	Pos	Pos	Neg	633	129	1
WWTP (1g)	5855	>32	12	3	0.25	0.023	0.25	1	0.125	Pos	Pos	Neg	651	239	1
WWTP (1i)	5856	>32	>256^*a*^	>256^*a*^	0.25	2	0.75	2	0.38	Pos	Pos	Neg	012	54	1
WWTP (1j)	5857	>32	4	2	0.38	0.032	0.75	0.75	0.38	Pos	Pos	Neg	014	13	1
WWTP (2a)	5858	24	8	>256	0.38	4	0.5	1	0.75	Pos	Pos	Pos	078	11	5
WWTR (2b)	5859	6	16	>256	0.5	4	0.75	1	0.5	Pos	Pos	Pos	078	11	5
Lake (4e)	5860	>32	12	6	0.38	0.048	0.38	1	0.38	Pos	Pos	Neg	002	8	1
River1_PWTP (5h)	5861	>32	>256^*a*^	>256^*a*^	0.38	0.032	0.25	1	0.25	Neg	Neg	Neg	009	3	1
River1 (9d)	5862	>32	>256^*a*^	>256^*a*^	0.38	2	0.75	1.5	0.094	Neg	Neg	Neg	085	39	4
River1 (10f)	5863	>32	>256^*a*^	>256^*a*^	0.19	0.023	0.75	2	12^*b*^	Neg	Neg	Neg	010	15	1
River2 (11)	5864	>32	8	3	0.38	<0.016	0.25	1	<0.016	Pos	Pos	Neg	014	2	1
River2 (12b)	5865	>32	16	0.75	0.5	1	0.25	1	0.19	Pos	Pos	Neg	043	103	1

a *ermB* gene detected. ST, sequence type; CIP, ciprofloxacin; CLD, clindamycin; ERY, erythromycin; MTZ, metronidazole; TET, tetracycline.

b MIC in first culture of isolate was 256 mg/L.

**FIG 2:**
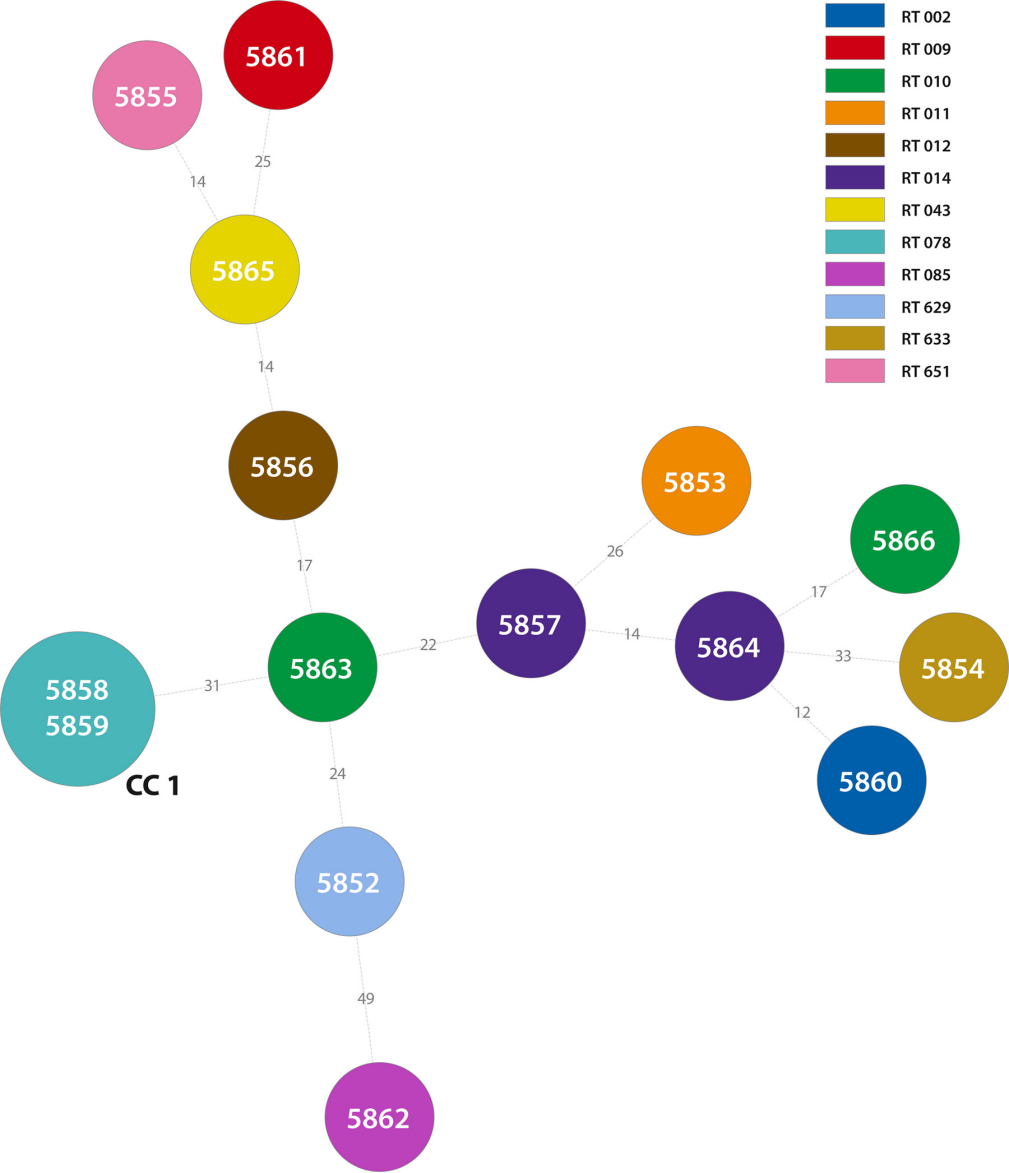
A minimum-spanning tree of Clostridioides difficile isolates cultured from wastewater and surface water samples constructed from multilocus variable-number tandem-repeat analysis (MLVA) results. The numbers in circles represent the DNA number of C. difficile isolates. The numbers on the lines represent the sum of tandem repeat differences (STRD) between isolates. If more than one number is present in one circle, it represents isolates with STRD of 0. Each ribotype is represented in a different color.

The molecular typing of 15 C. difficile isolates showed 12 different ribotyping profiles and 14 sequence types (STs), respectively (Table 2; Supplemental material). The STs identified clustered into three clades (1, 4, and 5), with clade 1 being the most represented (11 STs) (Table 2). Using multilocus variable-number tandem-repeat analysis (MLVA), two isolates of ribotype (RT) 078 had an identical number of tandem repeats in six variable-number tandem-repeat (VNTR) loci investigated, and two isolates of RT010 and RT014 were unrelated by analysis of seven VNTR loci (Fig. 2).

Five isolates of four RTs (009, 010, 085, 629) did not carry any of the toxin genes; two isolates of RT078 carried genes for toxins A, B, and binary. The remaining eight C. difficile isolates of seven RTs (002, 011, 012, 014, 043, 633, and 651) carried genes for toxins A and B (Table 1).

All C. difficile isolates were susceptible to amoxicillin, moxifloxacin, tetracycline, and vancomycin and resistant to ciprofloxacin. A high level of erythromycin resistance (>256 mg/L) was detected in eight isolates, six of which carried the *ermB* gene. Clindamycin resistance was found in 14 isolates; 6 isolates showed a high level of resistance (>256 mg/L) and carried *ermB* (Table 2). Surprisingly, one isolate (RT010, ST15) showed high resistance to metronidazole (>256 mg/L), but in subcultures, the MIC decreased to 12 mg/L. The presence of the recently published plasmid pCD-METRO (13) has been detected by PCR amplification and Sanger sequencing of 6 of 8 open reading frames (ORFs) (Supplemental material). The plasmid origin of metronidazole resistance was confirmed by PCR amplification of plasmid-specific amplification (targeting ORF6 and ORF3) and the absence of chromosomal-specific amplification (*gluD*) after PlasmidSafe DNase treatment (supplemental material).

## DISCUSSION

C. difficile is a major pathogen of gastrointestinal infection associated with health care; however, given its occurrence outside health care settings, there is a need to understand the epidemiology of CDI, and therefore, the One Health Concept of research has to be performed. In the Czech Republic, the CDI epidemiology in health care settings has been mapped in several studies, and the predominance of RTs 001 and 176 is persistent (14); however, data from community CDIs are lacking. Recent Czech data on livestock (piglets, sows, and calves) and horses have been reported (15–17), and hospital-predominant lineages were not identified. In the present study, water samples from two rivers and a lake and wastewater treatment plant and samples from fish and gulls have been investigated for the presence of C. difficile.

In our study, six C. difficile isolates were cultured from surface water; culture positivity was 6 out of 14, or 42.8%. More samples from surface water samples (river, lake) were surprisingly positive by direct culture of the filters than after selective enrichment with alcohol shock (5 samples versus 1 sample). Zidaric et al. also studied the occurrence of C. difficile in river water and compared three methods of detection, including culture on selective agar with and without ethanol shock and real-time PCR. In contrast to our study, culture on selective media after alcohol shock was the most sensitive method for the detection of C. difficile (18).

Both two WWTP samples from two sampling days were culture positive. On the first sampling day, seven different ribotypes were detected by direct culture, but on the second sampling day, the culture was positive only after selective enrichment. In the study of Steyer et al., which is similar to our study, the direct culture of filters on selective agar yielded successful C. difficile culture positivity from WWTP samples (19). It should be noted that compared to the Slovenian study, in our study, the water samples were not heat treated before filtration, which could negatively affect the detection of C. difficile, especially with more contaminated samples from WWTPs. Interestingly, the authors Romano et al. used only selective enrichment to examine samples from WWTPs, and ribotype 078 also dominated among the detected ribotypes (20). Here, it is possible to argue about whether one ribotype can overgrow others during selective enrichment, which may change the actual representation of ribotypes in the sample. However, when comparing the culture positivity by direct plating and using enrichment cultures, the statistical significance was not reached (*P* = 0.289, McNemar’s test), but the combination of two approaches yielded two additional positive samples (3 isolates).

Six different ribotypes and sequence types were identified from the surface water samples, although half of the C. difficile isolates did not carry any toxin genes. Similar great diversity and the presence of nontoxigenic and toxigenic ribotypes were also found in Slovenian and Australian water samples (18, 21). The study from Slovenia investigated water from 25 rivers, and C. difficile was detected in at least 1 sample from 17 rivers (68.0%). A total of 154 C. difficile isolates were cultured; 110 (71.4%) of them were toxigenic, and 44 (28.6%) were nontoxigenic. A study from Australia cultured C. difficile from 47.3% (53/112) of lake/pond, 23.0% (14/61) of the river, and 20.0% (3/15) of estuary samples. In both studies, the toxigenic RT014 was the most common type, accounting for 25 isolates from 11 sampling sites in Slovenian and 10.5% (8/76) in the Australian study (18, 21). Interestingly, whole-genome sequencing of RT014 and RT020 Australian isolates of water origin and 26 clinical RT014/RT020 isolates revealed five groups with ≤10 core-genome single nucleotide polymorphisms (SNPs) that comprised human and water strains (21), and three different sequence types (STs), 2, 13, and 49, respectively, were identified (21). In our study, only one isolate from the river sample belonged to RT014 and ST2.

Interestingly, resistance to metronidazole, a drug still used for the treatment of CDI in humans (22) and acute diarrhea in dogs (23), was detected in one isolate (5863, 10f) from river samples. Recently, the authors Boekhoud et al. reported that metronidazole resistance correlates with the presence of a 7-kb plasmid, pCD-METRO (13). We used the primers published in their study and the presence of 6 ORFs of published pCD-METRO, and their locations in the plasmid DNA were confirmed. Our isolate belonged to RT010, which is in concordance with the study of Boekhoud et al., where the majority of isolates showing metronidazole resistance belonged to this ribotype (13). Surprisingly, one of the human RT010 isolates from the study of Boekhoud et al. was also of Czech origin (13). As RT010 is a nontoxigenic ribotype, it cannot cause CDI; however, the possible transmissibility of pCD-METRO and metronidazole resistance phenotype has been suggested (13).

The plasmid contains a small pseudogene with protein homology to the *nimB* gene from Bacteroides fragilis (13). The *nimB* gene has also been demonstrated in other species of anaerobes related to B. fragilis (24). It can be assumed that horizontal transfer can occur in the gut of hosts or, alternatively, during anaerobic wastewater treatment processes.

Contaminated surface water and biosolids from wastewater could also be a potential source of C. difficile colonization of wild animals. Rodriguez-Palacios et al. assumed that aquatic birds could be contaminated by C. difficile spores from water sources and could spread them (10). In our study, C. difficile was not detected in samples from juvenile gulls, a finding that correlates with the Slovenian study of 465 passerine birds during their migration south over the Alps (25). Similarly, we have not detected C. difficile in the intestinal content of the fish, in contrast to the study on fish in retail from Canadian grocery stores (12).

Wastewater samples were previously investigated in several studies. In our study, a total of nine C. difficile isolates of eight ribotypes and sequence types were cultured from WWTP samples. Two C. difficile isolates were nontoxigenic, and binary toxin genes were detected in two isolates of RT078. However, MLVA suggested that isolates of RT078 are genetically related (Fig. 2). A similar great diversity of ribotypes was found in the studies investigating WWTP samples from New Zealand, where 10 C. difficile isolates of 8 ribotypes were cultured (26). The study from southern Switzerland, which looked at 6 WWTPs, identified 13 different reference PCR ribotypes in 47 C. difficile isolates, of which the most common was RT078 (40%) (20). In Italy, the wastewater contained clinically significant ribotypes such as 078 (13 isolates); 014, 020, and 077 (8 isolates); 126 (6 isolates); and 011 and 018 (4 isolates) (27). Whole-genome sequencing (WGS) of isolates from wastewater from the east of England identified 38 STs and, importantly, 5 pairs of highly similar isolates (≤2 SNPs different in the core genome) in clinical and wastewater collections (8). In Slovenia, samples from WWTP were collected in a year, and C. difficile was detected in all samples; 121 strains were cultured, and 32 different ribotypes were identified with the predominance of RTs 014, 020, and 010 (19). In contrast, the Iranian study investigated 72 samples from WWTPs collected in Tehran over a year, and only one C. difficile isolate of ribotype 078 (using *slpA* typing) was identified; however, this isolate was found to be metronidazole resistant (11).

Although the study is limited by the small number of samples, several C. difficile ribotypes identified in our study from surface and wastewater overlap C. difficile ribotypes identified in previous studies carried out in the Czech Republic, i.e., from horses (009, 010, 012) and pigs (002, 011, 014, and 078) (15, 17), and, importantly, these were also cultured from hospitalized patients with CDIs (002, 011, 012, 014, 078, and 043), with the highest frequency for RTs 014 (8.1%) and 012 (5.8%) (28). The latest Czech study, including antimicrobial susceptibility data from human isolates, reported 19 isolates that revealed reduced susceptibility to metronidazole, but these belonged to epidemic ribotypes 001, 027, and 176, and the molecular mechanism was not investigated (28). However, human C. difficile isolates were derived from hospitalized patients and therefore do not reflect CDI epidemiology in the community.

### Conclusion.

A diverse spectrum of C. difficile strains was found in wastewater and surface water. Recently discovered plasmid-bound resistance to metronidazole was detected in C. difficile strain cultured from the surface water sample. Genomic comparative studies are needed to confirm the relatedness of these isolates with C. difficile isolates from animals and clinical CDI strains from humans.

## MATERIALS AND METHODS

### Study area and sample collection.

The study area is located within the South Moravia river basin in the lowlands of the southeastern part of the Czech Republic (Fig. 1) and was carried out between May and July 2019. Surface water samples were collected in the Nové Mlýny Reservoir (*n* = 2, near the nesting colony of gulls, from which cloacal swabs were taken) and its tributaries, the Svratka River (river 1) at six randomly selected locations, one before the WWTP and the remaining five downstream at different distances from WWTPs, and Jihlava River (river 2), with six randomly selected sampling points on its lower course that differed from the first river by the catchment area (medium-sized cities on the upper course) (Fig. 1; Table 1) (29). The catchment area of the reservoir is predominantly used for agriculture, but there is a large-sized town, Brno, and several middle-sized towns nearby. Treated wastewater was sampled twice a week apart at the outflow of a WWTP located at Brno from which the effluents go directly into the Svratka River (river 1) and further downstream into the Nové Mlýny Reservoir (Fig. 1; Table 1). All water samples were collected without sediment. Subsequently, samples of intestinal contents from standard-size caught fish, including breams (Abramis brama, *n* = 32) and asps (Leuciscus aspius, *n* = 5) from the one-off catch of consumer fish by a commercial company for retail, and cloacal swabs from nestling Caspian gulls (Larus cachinnans, *n* = 45) on the Nové Mlýny Reservoir were taken (Fig. 1; Table 1). Sampling dates and GPS coordinates are provided in Table 1.

### Clostridioides difficile culture.

Treated wastewater and surface water were sampled with the collection procedure recommended for standard water microbiological examination (30) into sterile 500-mL octagonal polyethylene terephthalate (PET) bottles (Corning, USA). Before culture for C. difficile, samples were filtered by the membrane filtration method (classic glass filter holder kit; Millipore), passing 100-mL volumes of each water sample through a 0.22-μm-pore-size cellulose ester membrane filter (Merck Millipore). One of each membrane was placed onto ChromID C. difficile selective agar (bioMérieux) and incubated anaerobically for up to 5 days. For the detection of C. difficile present in lower numbers, enrichment cultures were performed on all water samples by placing the second membrane of 100 mL filtrate into 50 mL cycloserine-cefoxitin fructose broth (CCFB; Oxoid) supplemented with 0.1% sodium taurocholate (Sigma-Aldrich) and incubated at 37°C for 8 days in anaerobic conditions (Concept 300; Ruskin). In the same way, the samples of intestinal contents from each caught fish (approximately 0.5 g) and cloacal swabs from nestling gulls were selectively enriched in 5 mL of CCFB. Thereafter, 1 mL of enriched sample and 1 mL of absolute ethanol were mixed and left for 1 h under occasional agitation at room temperature. Finally, tubes were centrifuged at 1,520 × *g* for 10 min, the supernatants were discarded, and the deposit was collected using sterile cotton-tipped swabs and plated onto the solid selective media described above. Inoculated plates from direct plating and after the enrichment were incubated under anaerobic conditions for 48 h at 37°C.

Inoculated plates that were negative after 48 h were incubated for a further 72 h before being discarded. All individual C. difficile colonies per plate were subcultured on Columbia blood agar (Oxoid), and subcultures were identified by matrix-assisted laser desorption ionization–time of flight mass spectrometry (MALDI-TOF MS) using a MALDI Biotyper v3.0 system (Bruker Daltonics). The isolates from the same sample were indexed with the same number and lowercase letters (Table 2) and preserved in a cryoprotective medium at −80°C until further processing.

### Antimicrobial susceptibility testing and detection of antibiotic resistance determinants.

The antimicrobial susceptibility testing of C. difficile isolates to amoxicillin, enrofloxacin, clindamycin, erythromycin, metronidazole, moxifloxacin, tetracycline, and vancomycin was performed using Etest (bioMérieux) on Brucella blood agar (Oxoid) containing hemin (5 μg/mL), vitamin K_1_ (10 μg/mL). The MIC breakpoints for metronidazole, vancomycin, and moxifloxacin were applied as recommended by the European Committee on Antimicrobial Susceptibility Testing (EUCAST) (31). The MIC breakpoints for amoxicillin, clindamycin, and tetracycline were determined according to the Clinical and Laboratory Standards Institute guidelines (CLSI) of breakpoints for susceptibility testing of anaerobic bacteria (32). Due to the lack of a recommended breakpoint for erythromycin, the same breakpoint as for clindamycin was applied (Table 2).

The presence of antimicrobial-resistance determinants of clindamycin/macrolides (*ermB*) and metronidazole (pCD-METRO) were investigated with primers published before (13, 33) (supplemental material). Six PCR amplicons of the CD-pMETRO ORFs were sequenced using Sanger sequencing on a 3130xl genetic analyzer (Applied Biosystems) and mapped using Geneious software v11.2.6 to annotated genome assembly for IB136, including pCD-METRO (GenBank accession number CAADHH010000013) from the study of Boekhoud et al. (13, Supplemental material). To confirm the plasmid origin of metronidazole resistance, plasmid DNA was extracted using GenElute plasmid miniprep kit (Sigma) and treated by Plasmid-Safe ATP-dependent DNase to remove chromosomal DNA contamination (Lucigen). The gene-specific PCR targeting regions of ORF6, ORF3 (plasmid specific), and *gluD* (chromosomal specific) was performed with treated and nontreated DNA samples (Supplemental material).

### Capillary electrophoresis PCR ribotyping, multilocus sequence typing, and toxin gene detection.

The capillary electrophoresis (CE) PCR ribotyping was performed according to the consensus PCR ribotyping protocol (34, 35). The WEBRIBO database was used for PCR ribotype determination (36). Multilocus sequence typing (MLST) and MLVA were performed as described previously (37, 38). The toxin genes *tcdA* (toxin A), *tcdB* (toxin B), and *cdtA*/*cdtB* (binary toxin) were detected by multiplex PCR. The primers for C. difficile 16S rRNA were used as an internal control for the PCR amplification for nontoxigenic strains (39). Primers used in our study are listed in the supplemental material.

### Ethical approval statement.

A sampling of Caspian gulls was authorized by the local Czech nature protection authorities (permissions S-JMK78643/2018 OŽP/Ško and S-JMK 40970/2019 OŽP/Ško). Samples from the fish were obtained at a local fish processing plant by the water reservoir; thus, ethical approval for these samples was not required.
