# Supplementary material for: Detection of Plasmid-Mediated Resistance to Metronidazole in Clostridioides difficile from River Water
Source: Microbiol Spectr. 2022 Aug 11;10(4):e00806-22. doi: 10.1128/spectrum.00806-22 (PMC9431275; doi:10.1128/spectrum.00806-22)

Primers used in the study

pCD-METRO (metronidazole resistance)

			Study
oBH-1	CCTCGTAGAATCCGGTGCAA	Forward primer annealing to orf6 of pCD-METRO	Boekhoud et al., 2020
oBH-2	TATTTCCTTGCCGCTGAGG	Reverse primer annealing to orf6 of pCD-METRO	Boekhoud et al., 2020
oBH-3	GCAGAGCGTTGTGGTATTC	Forward primer annealing to orf6 of pCD-METRO	Boekhoud et al., 2020
oBH-4	GTATTTCCTTGCCGCTGAG	Reverse primer annealing to orf6 of PCD-METRO	Boekhoud et al., 2020
oBH-5	AGTAGGCGGTCGGTACTTCT	Forward primer annealing to orf5 of pCD-METRO	Boekhoud et al., 2020
oBH-6	CTTCCGTTCCGCTTGTTTGC	Reverse primer annealing to orf5 of pCD-METRO	Boekhoud et al., 2020
oBH-7	GGCTCAGACACTTCTACCGC	Forward primer annealing to orf7 of pCD-METRO	Boekhoud et al., 2020
oBH-8	CCCCTTCCAGGGTGTTTTCT	Reverse primer annealing to orf7 of pCD-METRO	Boekhoud et al., 2020
oBH-9	GGGCTATAAGACCGACTGGC	Forward primer annealing to orf3 of pCD-METRO	Boekhoud et al., 2020
oBH-10	AACGGTCTCTACCTCCGTCA	Reverse primer annealing to orf3 of pCD-METRO	Boekhoud et al., 2020
oBH-11	ACTTACACTGCAAACGGTGC	Forward primer annealing to orf8 of pCD-METRO	Boekhoud et al., 2020
oBH-12	TCGTTGCTTGTGAGGTGAG	Reverse primer annealing to orf8 of pCD-METRO	Boekhoud et al., 2020

Ribotyping

RBT_forwarc	FAM-GTGCGGCTGGATCACCTCCT	bases 1482–1501 of the 16S ribosomal RNA gene	Bidet et al., 1999
RBT_revers	CCCTGCACCCTTAATAACTTGACC	bases 1–24 of the 23S ribosomal RNA gene	Bidet et al., 1999

Toxin genes

tcdA_F3345	GCATGATAAGGCAACTTCAGTGGTA	tcdA-toxin A gene_forward	Persson et al., 2009
tcdA_R3969	AGTTCCTCCTGCTCCATCAAATC	tcdA-toxin A gene_revers	Persson et al., 2009
tcdB_F5670	CCAAARTGGAGTGTTACAAACAGGT	tcdB-toxin B gene_forward	Persson et al., 2009
tcdB-R6079A	GCATTTCTCCATTCTCAGCAAAGTA	tcdB-toxin B gene_reverse	Persson et al., 2009
tcdB-R6079B	GCATTTCTCCGTTTTCAGCAAAGT	tcdB-toxin B gene_reverse	Persson et al., 2009
cdtA-F739A	GGGAAGCACTATATTAAAGCAGAAG	cdtA-binary toxin gene_forward	Persson et al., 2009
cdtA-F739B	GGGAAACATTATATTAAAGCAGAAG	cdtA-binary toxin gene_forward	Persson et al., 2009
cdtA-R958	CTGGGTTAGGATTATTTACTGGACC	cdtA-binary toxin gene_reverse	Persson et al., 2009
cdtB-F617	TTGACCCAAAGTTGATGTCTGATT	cdtB-binary toxin gene_forward	Persson et al., 2009
cdtB-R878	CGGATCTCTTGCTTCAGTCTTTATA	cdtB-binary toxin gene_reverse	Persson et al., 2009

***ermB*-gene (MLSB class antibiotic resistance)**

ermB-E5	CTCAAAACTTTTTAACGAGTG	23S ribosomal RNA methyltransferase_forward	Spigaglia et al,2004
ermB-E6	CCTCCCGTTAAATAATAGATA	23S ribosomal RNA methyltransferase_reverse	Spigaglia et al,2004

Multilocus tandem repeats analysis (MLVA)

A6Cd_F	FAM-TTAATTGAGGGAGAATGTTAA	Forward	van den Berg et al., 2007
B7Cd_F	FAM-CTTAATACTAAACTAACTCTAACCAGTAA	Forward	van den Berg et al., 2007
C6Cd_F	HEX-GTTTAGAATCTACAGCATTATTTG	Forward	van den Berg et al., 2007
E7Cd_F	FAM-TGGAGCTATGGAAATTGATA	Forward	van den Berg et al., 2007
F3Cd_F	HEX-TTTTTGAAACTGAACCAACAT	Forward	van den Berg et al., 2007
G8Cd_F	NED-TGTATGAAGCAAGCTTTTTAT	Forward	van den Berg et al., 2007
H9Cd_F	VIC-GTTTTGAGGAAACAAACCTATG	Forward	van den Berg et al., 2007
A6Cd_R	AAATACTTTTCCCACTTTCATAA	Reverse	van den Berg et al., 2007
B7Cd_R	TTATATTTTATGGGCATGTTAA	Reverse	van den Berg et al., 2007
C6Cd_R	ATTGGAATTGAATGTAACAAA	Reverse	van den Berg et al., 2007
E7Cd_R	CAAATACATCTTGCATTAATTCT	Reverse	van den Berg et al., 2007
F3Cd_R	ACAAAAGACTGTGCAAATATACTA	Reverse	van den Berg et al., 2007
G8Cd_R	AATCCAGCAATCTAATAATCCA	Reverse	van den Berg et al., 2007
H9Cd_R	GATGAGGAAATAGAAGAGTTCA	Reverse	van den Berg et al., 2007

Multilocus sequence typing (MLST)

adk1F	TTACTTGGACCTCCAGGTG	adk_Forward	Griffiths et al., 2010
adk1R	TTTCCACTTCCTAAGGCTG	adk_Reverse	Griffiths et al., 2010
atpA1F	TGATGATTTAAGTAAACAAGCT	atpA_Forward	Griffiths et al., 2010
atpA1R	AATCATGAGTGAAGTCTTCTC	atpA_Reverse	Griffiths et al., 2010
dxr3F	GCTACTTTCCATTCTATCTG	dxr_Forward	Griffiths et al., 2010
dxr4R	CCAACTCTTTGTGCTATAA	dxr_Reverse	Griffiths et al., 2010
glyA1F	ATAGCTGATGAGGTTGGAG	glyA_Forward	Griffiths et al., 2010
glyA1R	TTCTAGCCTTAGATTCTTCAT	glyA_Reverse	Griffiths et al., 2010
recA2F	CAGTAATGAAATTGGGAGAAG	recA_Forward	Griffiths et al., 2010
recA2R	ATTCAGCTTGCTTAAATGGT	recA_Reverse	Griffiths et al., 2010
sodA5F	CCAGTTGTCAATGTATTCATTT	sodA_Forward	Griffiths et al., 2010
sodA6R	ATAACTTCATTTGCTTTTACAC	sodA_Reverse	Griffiths et al., 2010
tpi2F	ATGAGAAAACCTATAATTGCAG	tpi_Forward	Griffiths et al., 2010
tpi2R	TTGAAGGTTTAACACTTCCAC	tpi_Reverse	Griffiths et al., 2010

Suplementary material: Molecular data on *C. difficile* isolates cultured from water samples				
*Primers used to amplify tcdA are located upstream of the repetitive region in the 3´-end. The TcdA-negative strains due to 3´-end deletion revealed positive PCR amplification [Persoon et al., 2008, 2009].				
**Ribotype or Webribotype**	**Toxin genes* [Persson *et al* ., 2009]**	**CE-ribotype peak size (bp) [Bidet *et al* ., 1999]**	**CE-ribotyping profile<br>HotStart Taq mastermix, 15m@95°C[60s@95°C, 60s@57°C, 60s@72°C,]x26, 30m@72°C<br>POP7, 1200 LIZ, 36cm array length [Fawley *et al., 2015;* Bidet *et al.* , 1999]**	**MLVA profile**
**DNA 5860**<br>RT002<br>ST8	*tcdA, tcdB*	282.47<br>284.12<br>324.35<br>326.46<br>444.58<br>484.51<br>541.30<br>546.13	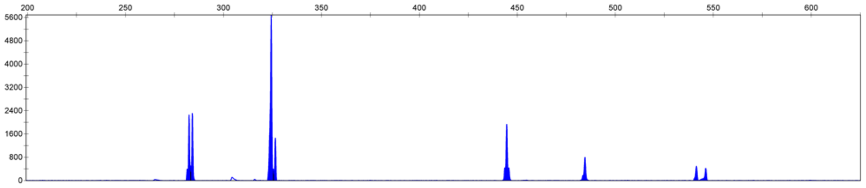	A6Cd: 34<br>B7Cd: 17<br>C6Cd: 36<br>E7Cd: 8<br>F3Cd: 6<br>G8Cd: 6<br>H9Cd: 1
**DNA 5861**<br>RT009<br>ST3	*non-toxigenic*	263.27<br>306.59<br>324.62<br>364.04<br>384.14<br>443.60<br>483.86<br>486.07<br>540.97	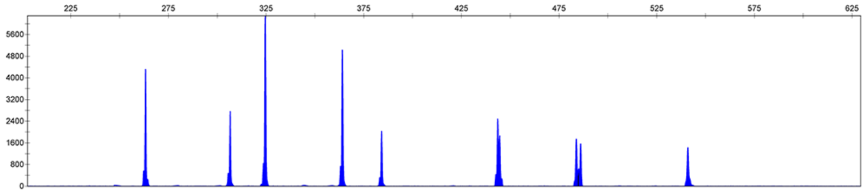	A6Cd: 29<br>B7Cd: 1<br>C6Cd: 30<br>E7Cd: 2<br>F3Cd: 6<br>G8Cd: 12<br>H9Cd: 2
**DNA 5863, 5866**<br>RT010<br>ST15	*non-toxigenic*	223.51<br>282.78<br>284.48<br>324.34<br>384.13<br>442.28<br>484.01<br>549.29	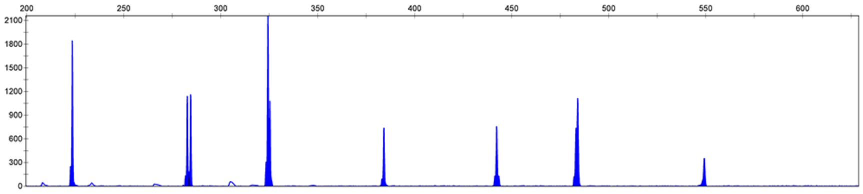	**5863**<br>A6Cd: 22<br>B7Cd: 14<br>C6Cd: 21<br>E7Cd: 7<br>F3Cd: 5<br>G8Cd: 10<br>H9Cd: 1<br>**5866**<br>A6Cd: 37<br>B7Cd: 14<br>C6Cd: 42<br>E7Cd: 8<br>F3Cd: 5<br>G8Cd: 10<br>H9Cd: 1
**DNA 5853**<br>RT011<br>ST325	*tcdA, tcdB*	234.13<br>264.08<br>284.33<br>324.19<br>365.46<br>443.71<br>487.77<br>540.78<br>542.53<br>546.44	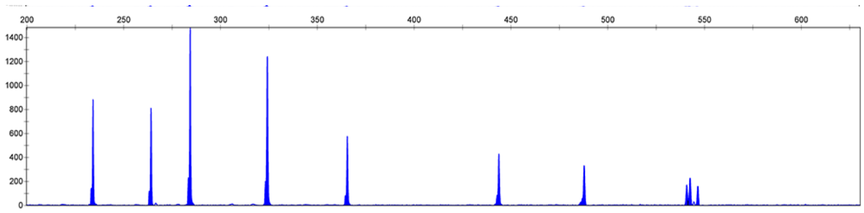	A6Cd: 14<br>B7Cd: 20<br>C6Cd: 45<br>E7Cd: 8<br>F3Cd: 6<br>G8Cd: 7<br>H9Cd: 2
**DNA 5856**<br>RT012<br>ST54	*tcdA, tcdB*	264.34<br>306.19<br>326.21<br>354.30<br>424.99<br>482.67<br>544.03	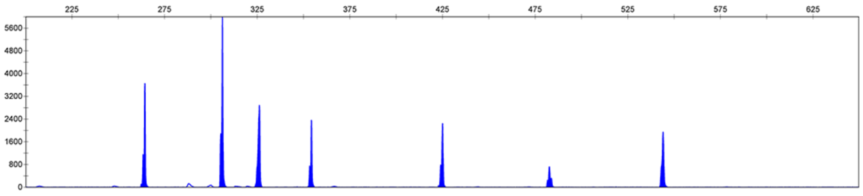	A6Cd: 24<br>B7Cd: 9<br>C6Cd: 20<br>E7Cd: 6<br>F3Cd: 7<br>G8Cd: 7<br>H9Cd: 4
**DNA 5857, 5864**<br>RT014<br>ST2	*tcdA, tcdB*	232.19<br>234.17<br>264.21<br>303.90<br>306.32<br>324.59<br>482.05<br>525.45<br>543.75	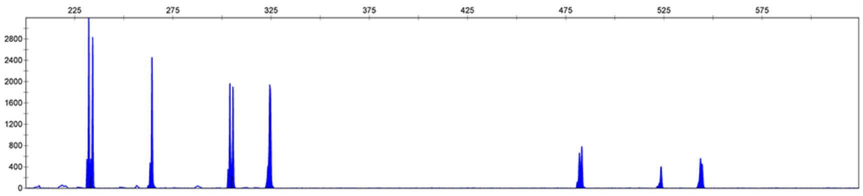	**5857**<br>A6Cd: 22<br>B7Cd: 13<br>C6Cd: 37<br>E7Cd: 6<br>F3Cd: 5<br>G8Cd: 7<br>H9Cd: 2<br>**5864**<br>A6Cd: 33<br>B7Cd: 12<br>C6Cd: 36<br>E7Cd: 6<br>F3Cd: 5<br>G8Cd: 8<br>H9Cd: 2

**DNA 5865**<br>RT043<br>ST103	*tcdA, tcdB*	232.36<br>262.84<br>265.00<br>284.89<br>304.33<br>323.89<br>445.09<br>481.71<br>545.80	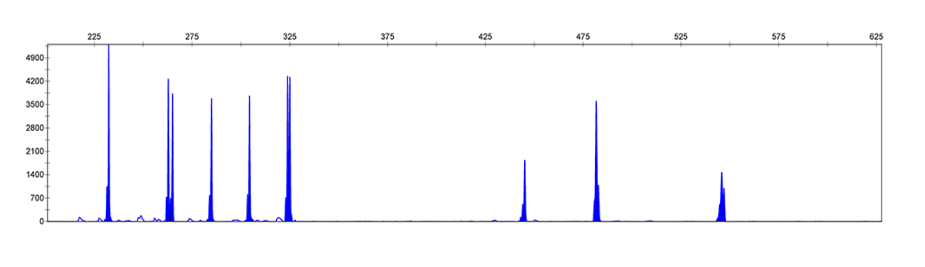	A6Cd: 23<br>B7Cd: 1<br>C6Cd: 20<br>E7Cd: 5<br>F3Cd: 5<br>G8Cd: 7<br>H9Cd: 2	
**DNA 5858, 5859**<br>RT078<br>ST11	*tcdA, tcdB, cdtA, cdtB 39bp*	304.28<br>324.78<br>327.28<br>368.52<br>370.76<br>412.64<br>443.12<br>486.48	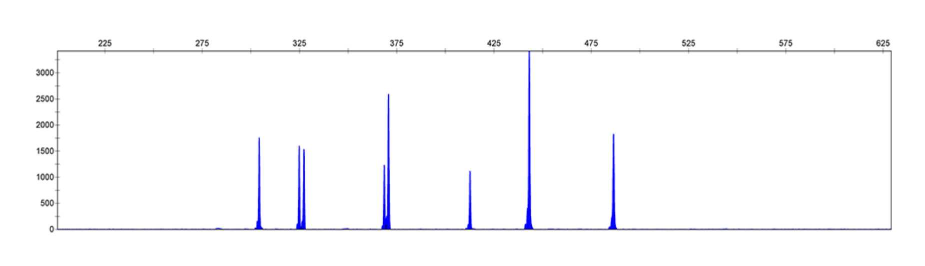	**5858**<br>A6Cd: NA<br>B7Cd: 17<br>C6Cd: 22<br>E7Cd: 5<br>F3Cd: 4<br>G8Cd: 11<br>H9Cd: 0	**5859**<br>A6Cd: NA<br>B7Cd: 17<br>C6Cd: 22<br>E7Cd: 5<br>F3Cd: 4<br>G8Cd: 11<br>H9Cd: 0
**DNA 5862**<br>RT085<br>ST39	*non-toxigenic*	232.32<br>264.9<br>324.7<br>345.96<br>446.6<br>546.9	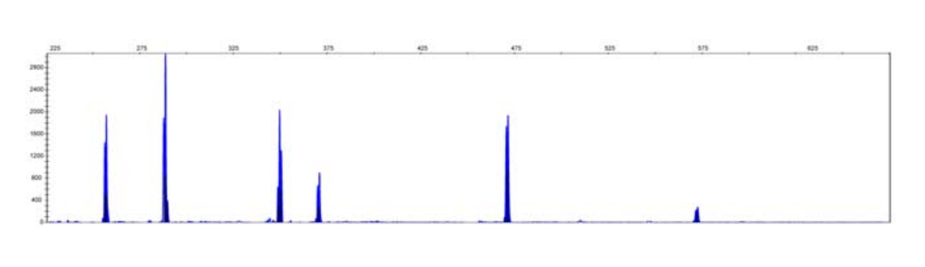	A6Cd: 33<br>B7Cd: 57<br>C6Cd: 23<br>E7Cd: 2<br>F3Cd: 5<br>G8Cd: 14<br>H9Cd: 1	
**DNA 5852**<br>RT629<br>ST109	*non-toxigenic*	231.32<br>264.7<br>323.54<br>325.81<br>423.75<br>546.43	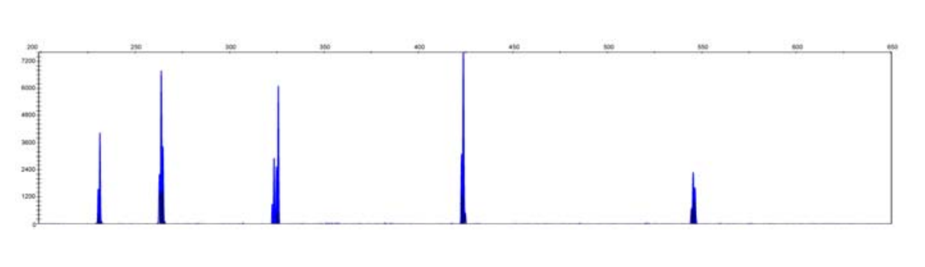	A6Cd: 23<br>B7Cd: 27<br>C6Cd: 20<br>E7Cd: 5<br>F3Cd: 6<br>G8Cd: 16<br>H9Cd: 1	
**DNA 5854**<br>RT633<br>ST129	*tcdA, tcdB*		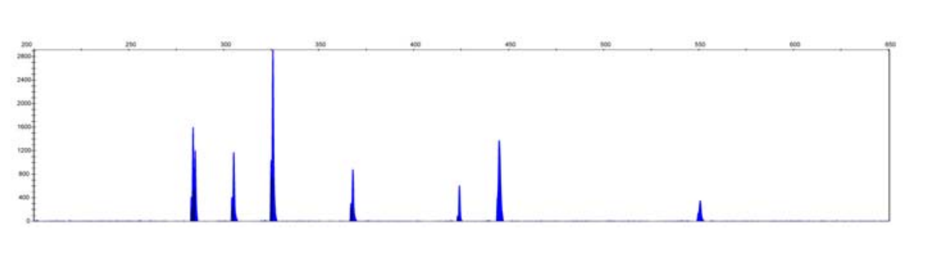	A6Cd: 46<br>B7Cd: 1<br>C6Cd: 37<br>E7Cd: 5<br>F3Cd: 7<br>G8Cd: 4<br>H9Cd: 1	
**DNA 5855**<br>RT651<br>ST239	*tcdA, tcdB*	232.36<br>283.89<br>324.94<br>325.89<br>368.12<br>426.18<br>444.91<br>550.72	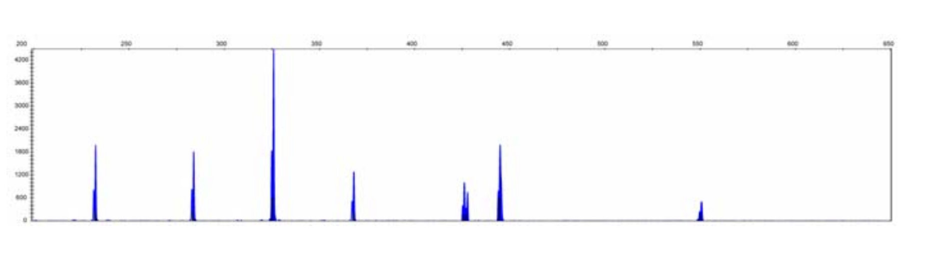	A6Cd: 26<br>B7Cd: 1<br>C6Cd: 15<br>E7Cd: 9<br>F3Cd: 4<br>G8Cd: 6<br>H9Cd: 2	

Six PCR amplicons of the CD-pMETRO ORFs were sequenced using Sanger sequencing on 3130xl Genetic Analyzer (Applied Biosystems) and mapped using Geneious software v11.2.6 to annotated genome assembly for IB136, including pCD-METRO (accession number CAADHH010000013 from the study Boekhoud et al., 2020

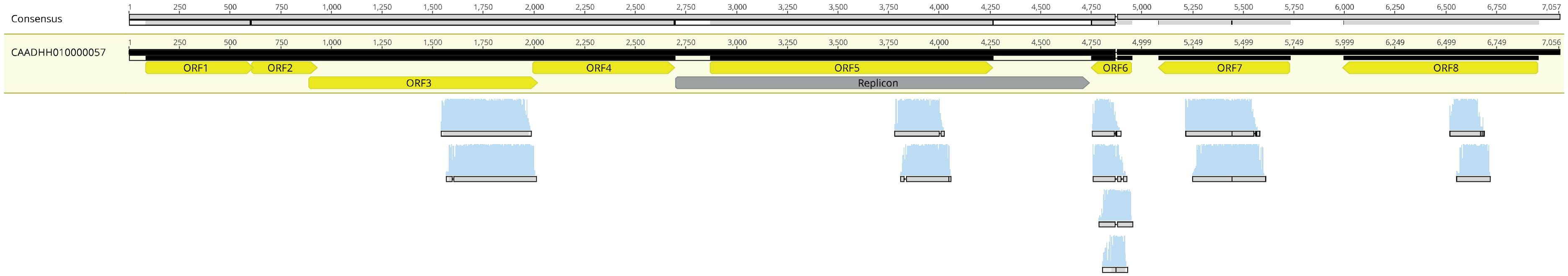

The products of plasmid-specific amplification (targeting ORF6 and ORF3) or chromosomal-specific amplification (*gluD*) before and after PlasmidSafe DNase treatment.

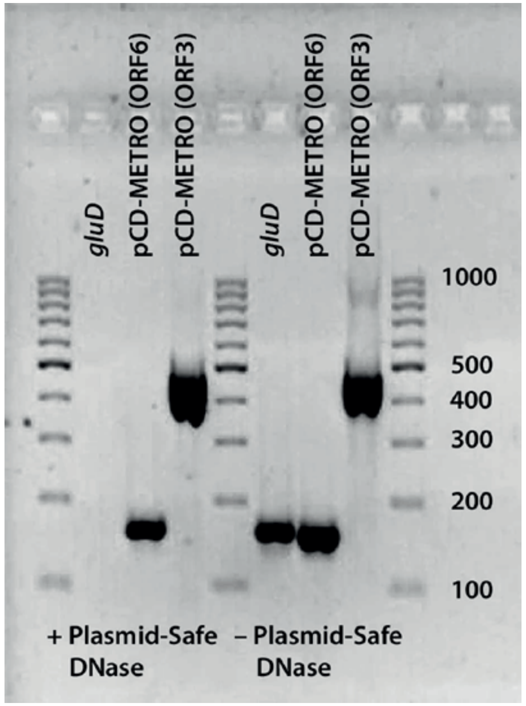

Supplement: Supplemental file 1 — Supplemental material. Download spectrum.00806-22-s0001.pdf, PDF file, 0.6 MB [file spectrum.00806-22-s0001.pdf]
